# Dominant mutations in *MIEF1* affect mitochondrial dynamics and cause a singular late onset optic neuropathy

**DOI:** 10.1186/s13024-021-00431-w

**Published:** 2021-02-25

**Authors:** Majida Charif, Yvette C. Wong, Soojin Kim, Agnès Guichet, Catherine Vignal, Xavier Zanlonghi, Philippe Bensaid, Vincent Procaccio, Dominique Bonneau, Patrizia Amati-Bonneau, Pascal Reynier, Dimitri Krainc, Guy Lenaers

**Affiliations:** 1 Université d’Angers, MitoLab team, UMR CNRS 6015 - INSERM U1083, Unité MitoVasc, Angers, France; 2 Genetics and Immuno-Cell Therapy Team, Mohammed First University, Oujda, Morocco; 3 Department of Neurology, Northwestern University Feinberg School of Medicine, Chicago, IL USA; 4 Departments of Biochemistry and Genetics, University Hospital Angers, Angers, France; 5 Neuroophthalmology Department, Rothschild Ophthalmologic Foundation, Paris, France; 6 Centre de Compétence Maladies Rares, Clinique Pluridisciplinaire Jules Verne, Nantes, France; 7 Cabinet d’Ophtalmologie, Morlaix, France

**Keywords:** Mitochondria dynamics, Mitochondrial disease, MIEF1, Mid51, Dominant optic atrophy (DOA), Inherited optic neuropathy (ION), Peripheral visual field, Neurodegeneration

## Abstract

Inherited optic neuropathies are the most common mitochondrial diseases, leading to neurodegeneration involving the irreversible loss of retinal ganglion cells, optic nerve degeneration and central visual loss. Importantly, properly regulated mitochondrial dynamics are critical for maintaining cellular homeostasis, and are further regulated by *MIEF1* (mitochondrial elongation factor 1) which encodes for MID51 (mitochondrial dynamics protein 51), an outer mitochondrial membrane protein that acts as an adaptor protein to regulate mitochondrial fission. However, dominant mutations in *MIEF1* have not been previously linked to any human disease. Using targeted sequencing of genes involved in mitochondrial dynamics, we report the first heterozygous variants in *MIEF1* linked to disease, which cause an unusual form of late-onset progressive optic neuropathy characterized by the initial loss of peripheral visual fields. Pathogenic *MIEF1* variants linked to optic neuropathy do not disrupt MID51’s localization to the outer mitochondrial membrane or its oligomerization, but rather, significantly disrupt mitochondrial network dynamics compared to wild-type MID51 in high spatial and temporal resolution confocal microscopy live imaging studies. Together, our study identifies dominant *MIEF1* mutations as a cause for optic neuropathy and further highlights the important role of properly regulated mitochondrial dynamics in neurodegeneration.

**Supplementary Information:**

The online version contains supplementary material available at 10.1186/s13024-021-00431-w.

## Main text

Inherited Optic Neuropathies (ION) are the most common mitochondrial diseases, leading to the irreversible loss of retinal ganglion cells, optic nerve degeneration and central visual loss [1, 2]. They can be maternally transmitted by the mitochondrial genome in the case of Leber hereditary optic neuropathy (LHON, MIM#535000), or by mono or bi-allelic autosomal mutations. In dominant optic atrophy (DOA, MIM165500), it is now well established that mutations in genes involved in mitochondrial dynamics such as *OPA1* (MIM#605290) are the main cause of the disease. More than 400 distinct pathogenic variants have been described in *OPA1* [3–6], affecting the pro-fusion activity of this large dynamin-related GTPase resulting in defective mitochondrial fusion [7, 8]. The implication of mitochondrial dynamics in DOA was further supported by the identification of dominant mutations in *MFN2* (MIM#608507) [9] and *OPA3* (MIM#606580) [10], two additional genes acting on mitochondrial dynamics.

More recently, we and others described cohorts of individuals affected by DOA caused either by mutations in *SPG7* (MIM#602783) or in *AFG3L2* (MIM#604581) [11–13], two genes encoding mAAA-proteases acting on mitochondrial fusion through the indirect control of OPA1 processing, and also involved in recessive hereditary spastic paraplegia type 7 [14] and dominant spino-cerebellar ataxia 28 [15], respectively. In addition, homozygous mutations in *YME1L1* (MIM#617302), which also disrupt OPA1 processing have been identified in individuals with optic atrophy and mitochondrial disorders [16]. Interestingly, *DNM1L* mutations resulting in excessive mitochondrial fusion have also been identified in patients with a similar ophthalmological presentation [17] (MIM603850), thus providing evidence that alterations of both fusion and fission compromise retinal ganglion cell survival. *DNM1L* encodes DRP1, another dynamin-related GTPase which requires a set of adaptor proteins including MID51, MID49, and MFF to be recruited on the mitochondrial outer membrane to exert and regulate its pro-fission activity [18–20]. So far, only biallelic nonsense *MFF* mutations (MIM#614785) have been identified in individuals with a severe encephalopathy associated with optic atrophy and peripheral neuropathy [21], while no mutation has been reported for optic atrophy in *MIEF1* or *MIEF2*, which encode for MID51 and MID49, respectively.

Together, these observations prompted us to screen all known genes involved in mitochondrial dynamics in molecularly undiagnosed ION cases. Here, we identify the first heterozygous pathogenic disease-causing mutations in *MIEF1*, found in individuals with non-syndromic late-onset ION characterized by initial loss of peripheral visual fields. Importantly, ION disease-linked *MIEF1* variants affect mitochondrial network dynamics, and ultimately highlight the crucial role of properly regulating this pathway in optic nerve physiology.

**Fig. 1:**
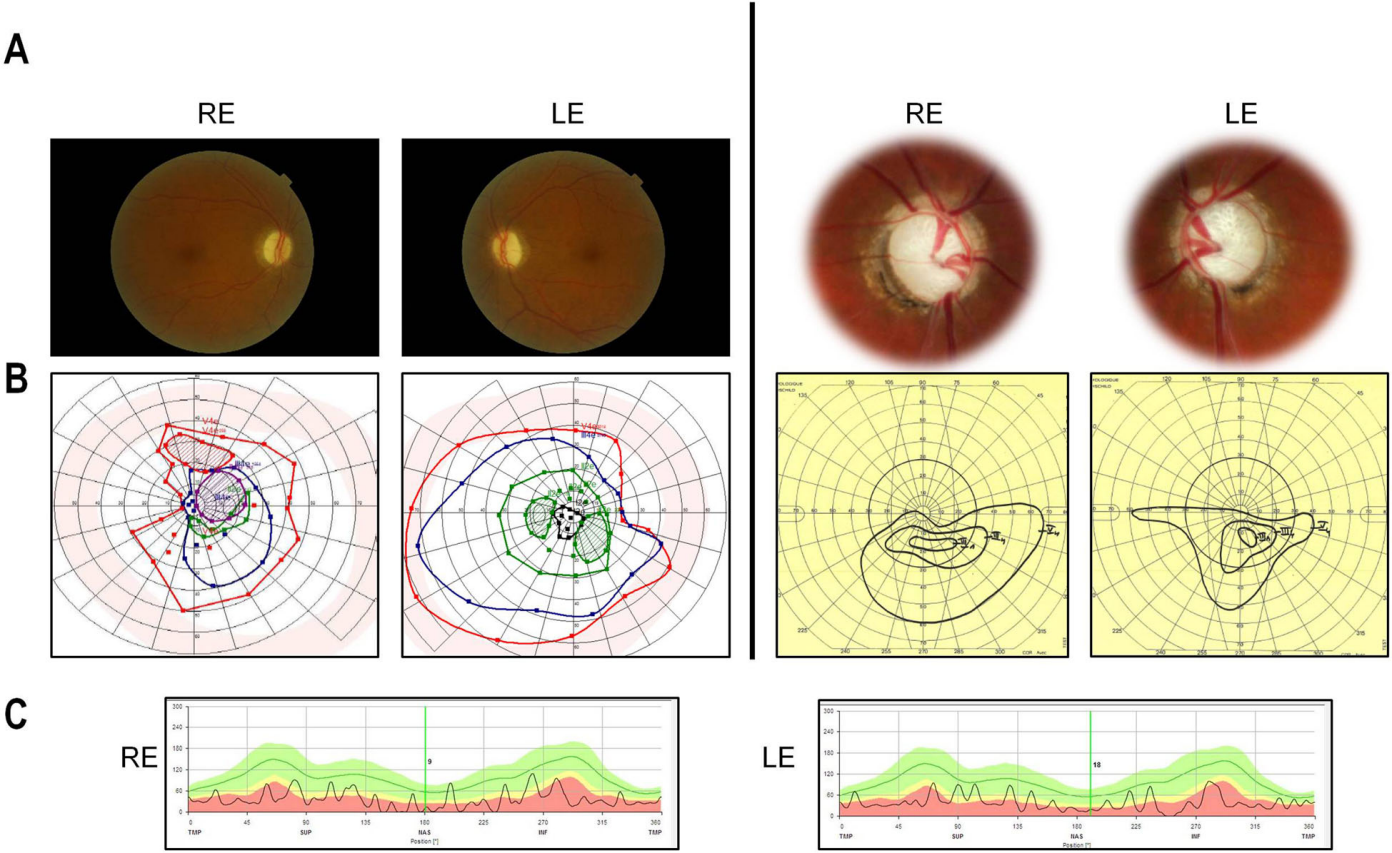
Characterization of inherited optic neuropathy patients with dominant *MIEF1* mutations. **a** Eye fundus of the right (RE) and left (LE) eye exhibiting severe optic disk pallor in *MIEF1* individuals with inherited optic neuropathy (Patient 1 (left 2 panels); Patient 2 (right 2 panels)). **b** Evaluation of the visual fields revealed peripheral visual field defects in *MIEF1* individuals with inherited optic neuropathy (Patient 1 (left 2 panels); Patient 2 (right 2 panels)). **c** Optical coherence tomography (OCT) recordings of the retinal nerve fiber layer (RNFL) at the papilla of the right (RE, left panel) and left (LE, right panel) eye from Patient 2 with inherited optic neuropathy

**Fig. 2:**
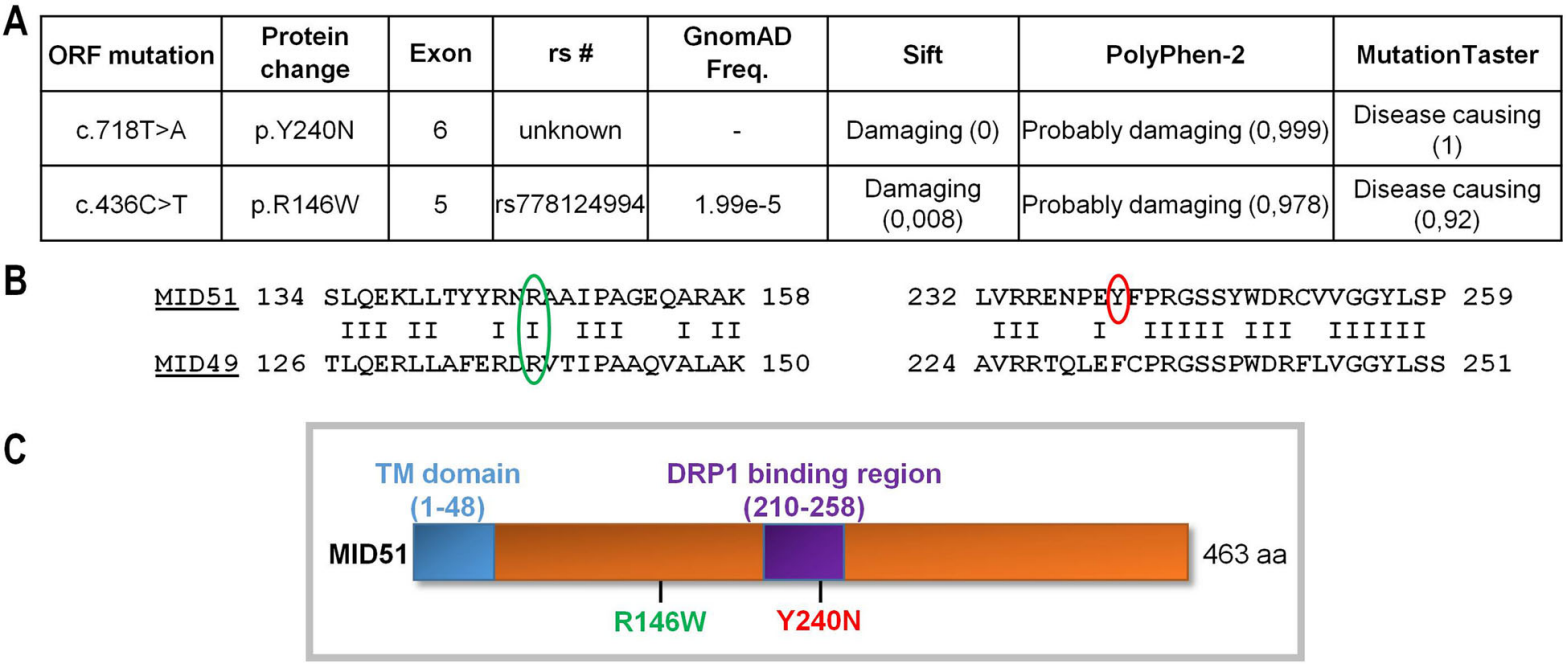
Genotype of dominant *MIEF1* variants in individuals with inherited optic neuropathy. **a** Genetic analysis identified dominant *MIEF1* mutations: heterozygous mutation c.718 T > A in exon 6 of *MIEF1* localized at position 240 of the MID51 protein, leading to an amino acid exchange of tyrosine to asparagine (p.Y240N; Patient 1); and heterozygous mutation c.436C > T in exon 5 of *MIEF1* localized at position 416 of the MID51 protein, leading to an amino acid exchange of arginine to tryptophan (p.R146W; Patient 2). Additional variant characterization according to the Sift, PolyPhen-2 and Mutation Taster scores are shown. **b** Alignment of MID51 (NM_019008.5) and MID49 (NM_139162.4) protein sequences around the mutated amino acids (p.R146W (green) and p.Y240N (red)) showing conservation of R146 in MID51. **c** Localization of the amino acid changes (p.R146W (green) and p.Y240N (red)) on the MID51 protein. MID51 contains a transmembrane domain (TM) mediating its insertion into the outer mitochondrial membrane, and a DRP1 binding region in which Y240 is located

Two hundred individuals in France affected by an ION were included in this study. None of the individuals included had a molecular diagnosis after screening for pathogenic variants in *OPA1*, *OPA3* and *WFS1* exonic sequences and LHON-associated mtDNA mutations. Individuals were analyzed using a targeted resequencing panel of 22 genes, including those already published as causing ION, and genes involved in mitochondrial dynamics (Supplementary Table 1). After eliminating frequent (allele frequency > 1/10.000) and non-pathogenic variants, according to prediction tools (Sift, Polyphen, and Mutation-Taster), we identified two individuals (Fig. 1) harboring a *MIEF1* heterozygous variant, which were confirmed by Sanger sequencing. The first individual harbored a c.718 T > A variant, not referenced in any database, and the second individual harbored a c.436C > T variant, referred to as rs778124994, with a frequency of 1.99e-5 in GnomAD database. Both variants were predicted to be damaging by the Sift and Polyphen programs and disease causing by Mutation Taster (Fig. 2a). These variants lead to the p.Y240N and the p.R146W amino-acid changes in MID51, respectively, with p.Y240N located in a DRP1 binding domain, and p.R146W located in another domain conserved within MID49, but without known function (Fig. 2b, c).

The two middle-aged women, 55 and 47 years old, carrying *MIEF1* mutations were initially referred to ophthalmology departments for a sudden painless visual acuity impairment (see Methods for detailed patient descriptions). At initial assessments, neither of the two individuals had symptoms or immunological profiles compatible with Glaucoma, Neuro Myelitis Optica or myelin oligodendrocyte glycoprotein optic neuritis. At fundus examination, both individuals had normal retina, but presented pale and moderately excavated optic disks (Fig. 1a). Importantly, both had an alteration of peripheral visual fields, with a clear-cut bilateral superior field defect in the second individual, together with variable central visual alterations (Fig. 1b). In both individuals, the disease progressed to poor vision (Patient 1: RE: 1/20, LE: 18/20; Patient 2: RE: hand moving, LE: 4/20), asymmetrically in the first one and symmetrically in the second one. Accordingly, optical coherence tomography (OCT) scanning disclosed severe retinal nerve fiber layer loss in all quadrants in the second individual (Fig. 1c). No additional neurological or systemic symptoms were observed in either patient.

Thus, the optic atrophy that we report here is an original ophthalmological presentation based on three discriminating criteria. First, the visual loss was noticed during adulthood, rather than during the first two decades, as commonly observed in other DOA, although a latent alteration of the peripheral visual fields might have preceded the loss of visual acuity by several years, as observed in one individual. Secondly, both patients complained of a rather sudden painless loss of visual acuity, similar to those observed in maternally inherited LHON, although without optic disk elevation or edema, which is distinct from what is generally observed for other autosomal optic neuropathies, except for a recent report presenting a LHON-like patient with bi-allelic *NDUFS2* mutations [22]. Thirdly, both cases examined here showed that the disease progressed from the peripheral to the central visual field, still preserving some visual acuity in the left eye of the first patient. This is in contrast with all the reports of ION cases, in which the alteration of the visual field occurs and evolves from the center to the periphery.

**Fig. 3:**
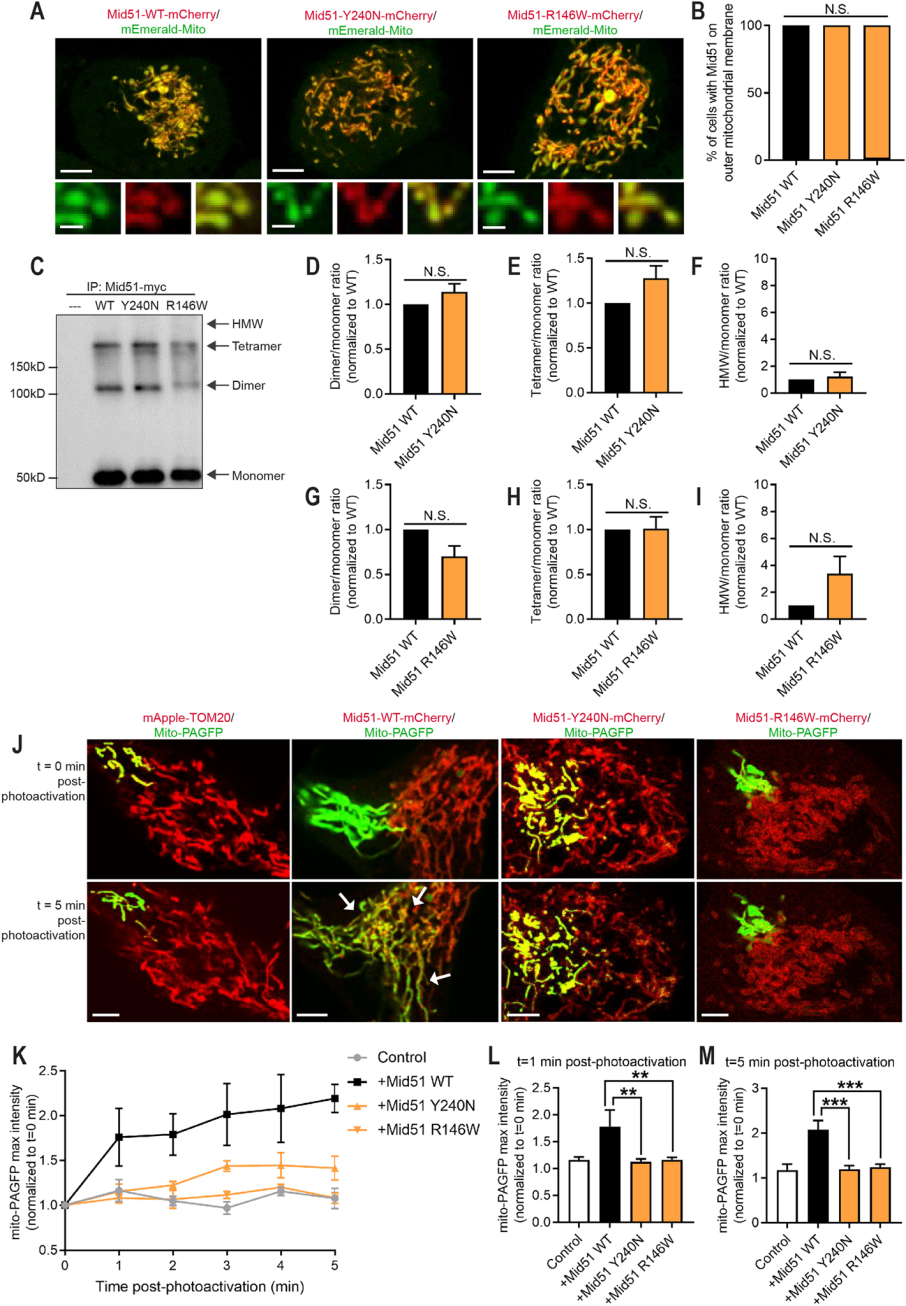
Inherited optic neuropathy MIEF1 mutations do not disrupt MID51 localization or oligomerization, but preferentially affect its ability to regulate mitochondrial network dynamics. **a** and **b** Representative live-cell confocal images and quantification of mCherry-tagged MID51 (WT; p.Y240N; p.R146W) showing MID51 localization to mitochondria (mEmerald-Mito). (*n* = 100 cells from 3 experiments/ per condition). Scale bar, 10 μm (inset, 1 μm). **c**-**i** Representative immunoblot (α-myc) and quantification of MID51 oligomerization into dimer, tetramer, or high molecular weight (HMW) species from IP (myc) of myc-tagged MID51 (WT; p.Y240N; p.R146W) and control (−-). Mutants MID51 p.Y240N (**d**-**f**) and MID51 p.R146W (**g**-**i**) show similar oligomerization compared to wild-type MID51 (WT). (*n* = 3 experiments/per condition). **j**-**m** Representative confocal live-cell images and traces (**j** and **k**) and analysis (**l** and **m**) of mito-PAGFP fluorescence intensity in distal region (10 μm from the site of mitochondrial photoactivation) in high spatial and temporal resolution confocal microscopy live imaging studies, showing increased mitochondrial fusion (white arrows) in wild-type MID51 (WT) condition which is not observed in mutant MID51 p.Y240N and p.R146W conditions. (*n* = 8 cells (control); *n* = 9 cells (WT); *n* = 16 cells (p.Y240N); *n* = 30 cells (p.R146W), from 3 experiments/per condition). Scale bar, 5 μm. Data are means ± s.e.m. (N.S. = not significant; **P < 0.01; ***P < 0.001; unpaired two-tailed t test (**d**-**i**); ANOVA with Tukey’s post-hoc test (**b**, **l**, **m**))

To assess the pathogenicity of the *MIEF1* variants, we expressed wild-type and both mutants (p.Y240N and p.R146 W) of MID51 in HeLa cells. MID51 is embedded in the outer mitochondrial membrane, where it homo-oligomerizes to regulate mitochondrial fission [18, 19]. Thus, we first examined whether p.R146W and p.Y240N mutants displayed a mitochondrial localization, using high spatial and temporal confocal microscopy in live cells. Expression of wild-type MID51 robustly localized to mitochondria (mEmerald-Mito) (Fig. 3a, b), consistent with its reported localization within the outer mitochondrial membrane [18, 19]. This was also true for mutant MID51 p.Y240N and p.R146W proteins, which also localized to the mitochondrial network in live cells (Fig. 3a, b), demonstrating that these *MIEF1* missense variants linked to optic neuropathy do not alter MID51’s mitochondrial localization.

We next investigated whether *MIEF1* variants disrupted the ability of MID51 to self-oligomerize. To test this, we immunoprecipitated myc-tagged wild-type and mutant MID51 proteins, and analyzed their oligomerization patterns. Wild-type MID51 formed monomers as well as oligomers, which consisted of dimers, tetramers, and very few high molecular weight (HMW) species (Fig. 3c). Similarly, we found that both mutant proteins MID51 p.Y240N (Fig. 3d-f) and MID51 p.R146W (Fig. 3g-i) also showed similar oligomerization patterns to wild-type MID51 (Fig. 3c), with similar ratios of each oligomeric species to monomer levels. Thus, these *MIEF1* missense variants linked to optic neuropathy do not significantly disrupt the oligomerization of MID51.

Finally, as MID51 is a known regulator of mitochondrial fission dynamics [18, 19], we studied whether mitochondrial fission/fusion dynamics were perturbed by the *MIEF1* variants. Expression of wild-type MID51 has previously been shown to inhibit mitochondrial fission, resulting in increased mitochondrial fusion events [19]. We analyzed the rate of mitochondrial fusion events in live cells, using a photo-activatable mitochondrial matrix probe (mito-PAGFP) in high spatial and temporal resolution confocal microscopy live imaging studies. A subpopulation of mitochondria was selectively photoactivated using a 405 nm laser, and the rate of fusion events with the surrounding mitochondria was analyzed by measuring the increase in fluorescence intensity over time at a distal region of 10 μm from the site of photoactivation (Fig. 3j). Consistent with previous reports, we found that wild-type MID51 expression increased the rate of fusion events (Fig. 3j-m) compared to control cells. In contrast, both MID51 p.Y240N and p.R146W mutants resulted in significantly decreased mitochondrial fusion events and disrupted mitochondrial network dynamics, as compared to wild-type MID51 (Fig. 3j-m). Together, these results show that *MIEF1* mutations linked to optic neuropathy preferentially disrupt the ability of MID51 to regulate mitochondrial fission/fusion dynamics.

In summary, combining our clinical, genetic and pathophysiological data, we identify the first known dominant *MIEF1* mutations linked to a human disease, resulting in a specific ophthalmological neurodegenerative disease. MID51 is a key regulator of mitochondrial dynamics [18, 19, 23–27]. Our findings that Mid51 optic neuropathy-linked variants disrupt mitochondrial fission/fusion dynamics but not its localization or oligomerization are consistent with the fact that p.Y240 is a residue located in the loop region (residues 238–242) critical for DRP1 binding [28, 29] which regulates mitochondrial fission [30]. In contrast, neither disease-linked mutations (p.Y240N or p.R146W) are located in MID51’s transmembrane domain which mediates its outer mitochondrial membrane localization (residues 1 to 48) [19], or in previously characterized residues mediating Mid51’s oligomerization [29]. Together, our work should prompt the molecular screening of *MIEF1* in ION individuals with severe alterations of the peripheral visual field, and further emphasizes the crucial role for properly regulated mitochondrial dynamics in neurodegenerative diseases.

## Methods

### Population screening

Two hundred individuals in France (112 males and 88 females) with an inherited optic neuropathy were included in this study. Their DNA samples were collected at the Department of Biochemistry and Genetics from the Angers University Hospital (France), which is a national centre for the molecular diagnosis of ION. All patients presented with an isolated dominant or recessive optic atrophy, and were diagnosed by an ophthalmologist from the French National Reference Centres for Rare Blinding Diseases.

### Subjects

The first affected individual aged 55 is from Maghreb origins (Patient 1). In 2002, she noticed a faint visual problem on the right eye, with a reported best visual acuity (bva) of 10/20 at first examination. In 2015, she complained of an acute painless visual loss, affecting mainly the peripheral visual field of both eyes, and also the central visual field of the right eye (bva:1/20), but not that of the left eye (bva: 18/20) (Fig. 1b, left). Visual evoked potentials were strongly affected for both eyes, while scotopic and photopic electroretinograms (ERGs) were normal. Eye fundus examination revealed a normal retina, while the optic disks appeared pale and moderately excavated (Fig. 1a, left). Blood analyses, and in particular vitamin B9 and B12 concentrations and Lyme and Treponema serology, were normal, but she had higher sedimentation rate (44 mm, with normal Protein C Reactive concentration). She also suffered from sero-negative rheumatoid polyarthritis treated by ARAVA (20 mg/day), while no peripheral neuropathy was noticed.

The second individual aged 47 was born in Egypt (Patient 2). She did not complain of any visual defect during her youth. In 2002, 1 month after an uneventful cesarean delivery, she complained of a painless visual loss first on the right eye (bva: 3/20), then on the left eye (bva: 10/20), while the visual field examination disclosed severely narrowed isopters, in particular on the superior quadrant, reflecting a bilateral superior altitudinal defect (Fig. 1b, right). She presented with pale and excavated optic disks (Fig. 1a, right) with normal intra-ocular pressure (IOP: RE: 9 and LE: 10 mmHg). Her bilateral optic neuropathy progressed to 2/20 and 8/20 in 2012, to moving hand and 4/20 in 2015, respectively for the right and left eye. VEP (visual evoked potential) were strongly affected, while the retina was normal, except for the presence of macular microcysts. OCT (optical coherence tomography) examination revealed collapsed RNFL (retinal nerve fiber layer) in all quadrants in both eyes (Fig. 1c). Anti-NMO and anti-MOG serology were negative. Brain MRI and ENT examinations were normal. She suffers from hypothyroidism, hypercholesterolemia and iron deficiency, but has no diabetes, epilepsy or neuro-muscular disorder. Biotinidase activity was normal.

In both individuals, Sanger sequencing did not reveal mutations in OPA1, OPA3, WFS1, or mtDNA LHON mutations. In addition, SNP array analyses revealed no chromosomal abnormality, no copy number variation and no large region of homozygosity that might have indicated a consanguinity.

### Standard protocol approvals, registrations, and patient consents

Written informed consent to perform genetic analyses was obtained from each subject involved in this study, according to protocols approved by the Ethical Committees of the different Institutes involved in this study, and in agreement with the Declaration of Helsinki (Institutional Review Board Committee of the University Hospital of Angers, Authorization number: AC-2012-1507).

### Genetic analysis

Genomic DNA was extracted from peripheral blood cells from a cohort of individuals in France with ION and screened for pathogenic variants in *OPA1*, *OPA3* and *WFS1* exonic sequences and the three primary LHON mtDNA mutations. Negative cases were analyzed by targeted resequencing panel of 22 genes (Supplementary Table 1) including published ION genes and candidate genes involved in mitochondrial dynamics (*FIS1, MFF, MFN1, MIEF1, MIEF2, OMA1* and *YME1L1*), but not known to be involved in ION.

The amplicon library targeting the exons from the 22 genes (Supplementary Table 1) was designed with the Ion AmpliSeq Designer (http://ampliseq.com). The Qubit dsDNA High Sensitivity Assay Kit (Thermo Fisher Scientific) was used to quantify DNA for NGS library construction. Library preparation for each sample was performed using Ion AmpliSeq technologies, sequencing was undertaken using 540 ChIPs on Ion S5™ Sequencer using barcoded samples, and sequencing data was processed using our own dedicated bioinformatics pipeline as described elsewhere [31, 32]. Within the design regions, 95% of bases had coverage over 100X, indicating that sufficient coverage was achieved to enable high variant detection sensitivity. Sanger sequencing was performed in parallel for regions that were not or poorly covered by ChIP sequencing.

For each patient, we screened for causative variants using the following prioritization strategy. Firstly, we checked reported pathogenic variants in ION published genes, then we looked for novel loss-of-function (LOF) variants (stop-gain, frameshift and splicing) or novel missense variants and finally, we selected pathogenic variants in candidate unpublished genes. All dominant variants were either absent or had a minor allele frequency (MAF) threshold of < 0.0001 in public databases (GnomAD), while recessive variants were considered with a MAF threshold < 0.005. For familial cases, we specifically selected the variants that matched the inheritance pattern predicted from the pedigrees [32]. The two candidate pathogenic variants in *MIEF1* were validated by Sanger sequencing.

### Reagents

The following plasmids were obtained from Addgene: mEmerald-Mito-7 was a gift from Michael Davidson (Addgene plasmid # 54160; http://n2t.net/addgene:54160; RID:Addgene_54,160) [33], mito-PAGFP was a gift from Richard Youle (Addgene plasmid # 23348; http://n2t.net/addgene:23348; RRID:Addgene_23,348) [34], mApple-TOMM20-N-10 was a gift from Michael Davidson (Addgene plasmid # 54955; http://n2t.net/addgene:54955; RRID:Addgene_54,955). C-terminal mCherry-tagged MID51 (wildtype (WT); p.Y240N; p.R146W) and C-terminal myc-tagged MID51 (wildtype (WT); p.Y240N; p.R146W) were generated by VectorBuilder. The following antibodies were also used: rabbit Myc-tag (Cell Signaling, 2272S) and mouse Myc-Tag (9B11) (Cell Signaling, 2276).

### Cell culture and transfection

HeLa cells (ATCC) were cultured in DMEM (Gibco; 11995–065) supplemented with 10% (vol/vol) FBS, 100 units per ml penicillin, and 100 μg/ml streptomycin. All cells were maintained at 37 °C in a 5% CO^2^ incubator and verified by cytochrome *c* oxidase subunit I (COI) and short tandem repeat (STR) testing, and were negative for mycoplasma contamination. Cells were transfected using Lipofectamine 2000 (Invitrogen). For live imaging, cells were grown on glass-bottom culture dishes (MatTek; P35G-1.5-14-C).

### Confocal microscopy

Confocal images were acquired on a Nikon A1R confocal microscope with GaAsp detectors using a Plan Apo λ 100 × 1.45 NA oil immersion objective (Nikon) using NIS-Elements (Nikon). Live cells were imaged in a temperature-controlled chamber (37 °C) at 5% CO_2_ at 1 frame every 2–3 s. For mito-PAGFP experiments, the matrix of a subpopulation of mitochondria were selectively labeled by localized photoactivation, using a 405 nm laser (100% for 4 s) in cells transfected with photoactivatable mitochondrial matrix marker mito-PAGFP and either control (mApple-Tom20) or mCherry MID51 (WT, Y240N or R146W). Fluorescence intensity at a distal region of 10 μm from the site of photoactivation was analyzed.

### Immunoprecipitation

To examine MID51 oligomerization, HeLa cells were transfected for 24 h with myc-tagged MID51 (WT, Y240N or R146W) or not transfected (control). Cells were lysed in EBC buffer (Boston Bioproducts; C14–10) with cOmplete*™* Protease Inhibitor Cocktail (Roche; 11,873,580,001) and sonicated. Lysates were immunoprecipitated using Protein G-coupled Dynabeads (Invitrogen; 100003D) incubated in anti-myc antibody (ms), and washed in EBC buffer. Equal total protein levels of immunoprecipitate were analyzed by SDS-PAGE and Western blot according to standard protocols using anti-myc antibody (Rb). Measured band intensities of immunoprecipitated MID51 for oligomeric species (dimer, tetramer or high molecular weight (HMW)) were normalized to monomeric MID51, and further normalized to MID51 (WT), and expressed as the ratio of MID51 oligomer/monomer for each condition.

### Image analysis

MID51 localization to mitochondria was quantified as the percentage of cells that had MID51 (mCherry-tagged) colocalized with mitochondria (mEmerald-mito). Mitochondrial fusion dynamics were analyzed as the mito-PAGFP fluorescence intensity at a distal region (10 μm from the site of photoactivation) at 1 min and 5 min post-photoactivation, and subsequently normalized to the intensity at t = 0 min. Example traces of mito-PAGFP max intensity (Fig. 3k) are the average of 3 independent examples (*n* = 3 cells from 3 experiments/ per condition). The mito-PAGFP fluorescence intensity used was the maximum intensity in a 4 μm × 4 μm area in the distal region. Immunoblots were quantified using ImageJ (NIH).

### Statistical analysis, graphing and figure assembly

Data were analyzed using unpaired two-tailed t test (for two datasets) and one-way ANOVA with Tukey’s post hoc test (for multiple datasets), and statistics are shown comparing MID51 (WT) to MID51 mutants. Data presented are means ± s.e.m. with *n* ≥ 3 independent experiments (biological replicates) per condition. Statistics and graphing were performed using Prism 7 (GraphPad) software. All videos and images were assembled using ImageJ 1.51j8 (NIH) and Illustrator CC (Adobe).

## Supplementary Information

**Additional file 1: Supplementary Table 1.** Targeted sequencing panel of ION genes and genes involved in mitochondrial dynamics.

## Data Availability

All data relevant to this study are contained within the article.

## Abbreviations

AFG3L2: AFG3 like matrix AAA peptidase subunit 2
Anti-MOG: Anti-myelin oligodendrocyte glycoprotein
Anti-NMO: Anti-neuromyelitis optica
DNA: Deoxyribonucleic acid
DNM1L/DRP1: Dynamin-related protein 1
DOA: Dominant optic atrophy
ENT: Ear, nose and throat
Fis1: Mitochondrial fission 1 protein
ION: Inherited Optic Neuropathies
IOP: Intra-ocular pressure
LHON: Leber hereditary optic neuropathy
MFF: Mitochondrial fission factor
MFN2: Mitofusin 2
MID49: Mitochondrial dynamics protein 49
MID51: Mitochondrial dynamics protein 51
MIEF1: Mitochondrial elongation factor 1
MIEF2: Mitochondrial elongation factor 2
mito-PAGFP: Mitochondrial photoactivatable green fluorescent protein
MRI: Magnetic resonance imaging
NDUFS2: NADH:Ubiquinone Oxidoreductase Core Subunit S2
OCT: Optical coherence tomography
OPA1: Optic Atrophy Protein 1
OPA3: Optic Atrophy Protein 3
RNFL: Retinal nerve fiber layer
SPG7: Spastic paraplegia 7
WFS1: Wolframin
WT: Wild type
YME1L1: YME1 Like 1 ATPase

## References

[1] Lenaers G, Hamel C, Delettre C, Amati-Bonneau P, Procaccio V, Bonneau D, Reynier P, Milea D (2012). Dominant optic atrophy. Orphanet J Rare Dis.

[2] Yu-Wai-Man P, Votruba M, Burte F, La Morgia C, Barboni P, Carelli V (2016). A neurodegenerative perspective on mitochondrial optic neuropathies. Acta Neuropathol.

[3] Alexander C, Votruba M, Pesch UE, Thiselton DL, Mayer S, Moore A, Rodriguez M, Kellner U, Leo-Kottler B, Auburger G (2000). OPA1, encoding a dynamin-related GTPase, is mutated in autosomal dominant optic atrophy linked to chromosome 3q28. Nat Genet.

[4] Delettre C, Lenaers G, Griffoin JM, Gigarel N, Lorenzo C, Belenguer P, Pelloquin L, Grosgeorge J, Turc-Carel C, Perret E (2000). Nuclear gene OPA1, encoding a mitochondrial dynamin-related protein, is mutated in dominant optic atrophy. Nat Genet.

[5] Ferre M, Caignard A, Milea D, Leruez S, Cassereau J, Chevrollier A, Amati-Bonneau P, Verny C, Bonneau D, Procaccio V, Reynier P (2015). Improved locus-specific database for OPA1 mutations allows inclusion of advanced clinical data. Hum Mutat.

[6] Le Roux B, Lenaers G, Zanlonghi X, Amati-Bonneau P, Chabrun F, Foulonneau T, Caignard A, Leruez S, Gohier P, Procaccio V (2019). OPA1: 516 unique variants and 831 patients registered in an updated centralized Variome database. Orphanet J Rare Dis.

[7] Olichon A, Landes T, Arnaune-Pelloquin L, Emorine LJ, Mils V, Guichet A, Delettre C, Hamel C, Amati-Bonneau P, Bonneau D (2007). Effects of OPA1 mutations on mitochondrial morphology and apoptosis: relevance to ADOA pathogenesis. J Cell Physiol.

[8] Bertholet AM, Delerue T, Millet AM, Moulis MF, David C, Daloyau M, Arnaune-Pelloquin L, Davezac N, Mils V, Miquel MC (2016). Mitochondrial fusion/fission dynamics in neurodegeneration and neuronal plasticity. Neurobiol Dis.

[9] Rouzier C, Bannwarth S, Chaussenot A, Chevrollier A, Verschueren A, Bonello-Palot N, Fragaki K, Cano A, Pouget J, Pellissier JF (2012). The MFN2 gene is responsible for mitochondrial DNA instability and optic atrophy ‘plus’ phenotype. Brain.

[10] Reynier P, Amati-Bonneau P, Verny C, Olichon A, Simard G, Guichet A, Bonnemains C, Malecaze F, Malinge MC, Pelletier JB (2004). OPA3 gene mutations responsible for autosomal dominant optic atrophy and cataract. J Med Genet.

[11] Klebe S, Depienne C, Gerber S, Challe G, Anheim M, Charles P, Fedirko E, Lejeune E, Cottineau J, Brusco A (2012). Spastic paraplegia gene 7 in patients with spasticity and/or optic neuropathy. Brain.

[12] Charif M, Roubertie A, Salime S, Mamouni S, Goizet C, Hamel CP, Lenaers G (2015). A novel mutation of AFG3L2 might cause dominant optic atrophy in patients with mild intellectual disability. Front Genet.

[13] Colavito D, Maritan V, Suppiej A, Del Giudice E, Mazzarolo M, Miotto S, Farina S, Dalle Carbonare M, Piermarocchi S, Leon A (2017). Non-syndromic isolated dominant optic atrophy caused by the p.R468C mutation in the AFG3 like matrix AAA peptidase subunit 2 gene. Biomed Rep.

[14] Casari G, De Fusco M, Ciarmatori S, Zeviani M, Mora M, Fernandez P, De Michele G, Filla A, Cocozza S, Marconi R (1998). Spastic paraplegia and OXPHOS impairment caused by mutations in paraplegin, a nuclear-encoded mitochondrial metalloprotease. Cell.

[15] Di Bella D, Lazzaro F, Brusco A, Plumari M, Battaglia G, Pastore A, Finardi A, Cagnoli C, Tempia F, Frontali M (2010). Mutations in the mitochondrial protease gene AFG3L2 cause dominant hereditary ataxia SCA28. Nat Genet.

[16] Hartmann B, Wai T, Hu H, MacVicar T, Musante L, Fischer-Zirnsak B, Stenzel W, Graf R, van den Heuvel L, Ropers HH (2016). Homozygous YME1L1 mutation causes mitochondriopathy with optic atrophy and mitochondrial network fragmentation. Elife.

[17] Gerber S, Charif M, Chevrollier A, Chaumette T, Angebault C, Kane MS, Paris A, Alban J, Quiles M, Delettre C (2017). Mutations in DNM1L, as in OPA1, result in dominant optic atrophy despite opposite effects on mitochondrial fusion and fission. Brain.

[18] Palmer CS, Osellame LD, Laine D, Koutsopoulos OS, Frazier AE, Ryan MT (2011). MiD49 and MiD51, new components of the mitochondrial fission machinery. EMBO Rep.

[19] Zhao J, Liu T, Jin SB, Wang XM, Qu MQ, Uhlen P, Tomilin N, Shupliakov O, Lendahl U, Nister M (2011). Human MIEF1 recruits Drp1 to mitochondrial outer membranes and promotes mitochondrial fusion rather than fission. EMBO J.

[20] Otera H, Wang C, Cleland MM, Setoguchi K, Yokota S, Youle RJ, Mihara K (2010). Mff is an essential factor for mitochondrial recruitment of Drp1 during mitochondrial fission in mammalian cells. J Cell Biol.

[21] Koch J, Feichtinger RG, Freisinger P, Pies M, Schrodl F, Iuso A, Sperl W, Mayr JA, Prokisch H, Haack TB (2016). Disturbed mitochondrial and peroxisomal dynamics due to loss of MFF causes Leigh-like encephalopathy, optic atrophy and peripheral neuropathy. J Med Genet.

[22] Gerber S, Ding MG, Gerard X, Zwicker K, Zanlonghi X, Rio M, Serre V, Hanein S, Munnich A, Rotig A (2017). Compound heterozygosity for severe and hypomorphic NDUFS2 mutations cause non-syndromic LHON-like optic neuropathy. J Med Genet.

[23] Koirala S, Guo Q, Kalia R, Bui HT, Eckert DM, Frost A, Shaw JM (2013). Interchangeable adaptors regulate mitochondrial dynamin assembly for membrane scission. P Natl Acad Sci USA.

[24] Loson OC, Song Z, Chen H, Chan DC (2013). Fis1, Mff, MiD49, and MiD51 mediate Drp1 recruitment in mitochondrial fission. Mol Biol Cell.

[25] Palmer CS, Elgass KD, Parton RG, Osellame LD, Stojanovski D, Ryan MT (2013). Adaptor proteins MiD49 and MiD51 can act independently of Mff and Fis1 in Drp1 recruitment and are specific for mitochondrial fission. J Biol Chem.

[26] Osellame LD, Singh AP, Stroud DA, Palmer CS, Stojanovski D, Ramachandran R, Ryan MT (2016). Cooperative and independent roles of the Drp1 adaptors Mff, MiD49 and MiD51 in mitochondrial fission. J Cell Sci.

[27] Kalia R, Wang RYR, Yusuf A, Thomas PV, Agard DA, Shaw JM, Frost A (2018). Structural basis of mitochondrial receptor binding and constriction by DRP1. Nature.

[28] Ma J, Zhai YJ, Chen M, Zhang K, Chen Q, Pang XY, Sun F (2019). New interfaces on MiD51 for Drp1 recruitment and regulation. PLoS One.

[29] Loson OC, Liu R, Rome ME, Meng SX, Kaiser JT, Shan SO, Chan DC (2014). The mitochondrial fission receptor MiD51 requires ADP as a cofactor. Structure.

[30] Richter V, Palmer CS, Osellame LD, Singh AP, Elgass K, Stroud DA, Sesaki H, Kvansakul M, Ryan MT (2014). Structural and functional analysis of MiD51, a dynamin receptor required for mitochondrial fission. J Cell Biol.

[31] Charif M, Chevrollier A, Gueguen N, Bris C, Goudenege D, Desquiret-Dumas V, Leruez S, Colin E, Meunier A, Vignal C (2020). Mutations in the m-AAA proteases AFG3L2 and SPG7 are causing isolated dominant optic atrophy. Neurol Genet.

[32] Felhi R, Sfaihi L, Charif M, Desquiret-Dumas V, Bris C, Goudenege D, Ammar-Keskes L, Hachicha M, Bonneau D, Procaccio V (2019). Next generation sequencing in family with MNGIE syndrome associated to optic atrophy: novel homozygous POLG mutation in the C-terminal sub-domain leading to mtDNA depletion. Clin Chim Acta.

[33] Planchon TA, Gao L, Milkie DE, Davidson MW, Galbraith JA, Galbraith CG, Betzig E (2011). Rapid three-dimensional isotropic imaging of living cells using Bessel beam plane illumination. Nat Methods.

[34] Karbowski M, Arnoult D, Chen H, Chan DC, Smith CL, Youle RJ (2004). Quantitation of mitochondrial dynamics by photolabeling of individual organelles shows that mitochondrial fusion is blocked during the Bax activation phase of apoptosis. J Cell Biol.

