# Supplementary material for: Dominant mutations in MIEF1 affect mitochondrial dynamics and cause a singular late onset optic neuropathy
Source: Mol Neurodegener. 2021 Feb 25;16:12. doi: 10.1186/s13024-021-00431-w (PMC7905578; doi:10.1186/s13024-021-00431-w)
Supplement: Supplementary file 1 — Additional file 1: Supplementary Table 1. Targeted sequencing panel of ION genes and genes involved in mitochondrial dynamics. [file 13024_2021_431_MOESM1_ESM.docx]

**Supplementary Table 1:**

**Targeted sequencing panel of ION genes and genes involved in mitochondrial dynamics.**

| **Gene** | **MIM#** | **Transcript** | **Function** |
| --- | --- | --- | --- |
| *ACO2* | **100850* | NM_001098 | Krebs cycle |
| *AFG3L2* | **604581* | NM_006796 | mito dynamics |
| *AOX1* | **602841* | NM_001159 | nucleotide metabolism |
| *CISD2* | **611507* | NM_001008388 | calcium homeostasis |
| *DNM1L* | **603850* | NM_012062 | mito dynamics |
| *FIS1* | **609003* | NM_016068 | mito dynamics |
| *MFF* | **614785* | NM_001277061 | mito dynamics |
| *MFN1* | **608506* | NM_033540 | mito dynamics |
| *MFN2* | **608507* | NM_014874 | mito dynamics |
| *MIEF1* | **615497* | NM_019008 | mito dynamics |
| *MIEF2* | **615498* | NM_139162 | mito dynamics |
| *NEFH* | **162230* | NM_021076 | cytoskeleton |
| *OMA1* | **617081* | NM_145243 | mito dynamics |
| *OPA1* | **605290* | NM_130837 | mito dynamics |
| *OPA3* | **606580* | NM_001017989 | mito dynamics |
| *PMPCA* | **613036* | NM_015160 | protein import to mito |
| *RTN4IP1* | **610502* | NM_032730 | Complex I assembly |
| *SLC25A46* | **610826* | NM_138773 | mito dynamics |
| *SPG7* | **602783* | NM_003119 | mito dynamics |
| *TMEM126A* | **612988* | NM_032273 | Complex I assembly |
| *WFS1* | **606201* | NM_006005 | calcium homeostasis |
| *YME1L1* | **607472* | NM_139312 | mito dynamics |
